# Risk factors of recurrence and life-threatening complications for patients hospitalized with chronic suppurative osteomyelitis of the jaw

**DOI:** 10.1186/1471-2334-13-313

**Published:** 2013-07-11

**Authors:** Lin Chen, Tangxin Li, Wei Jing, Wei Tang, Weidong Tian, Cai Li, Lei Liu

**Affiliations:** 1 Department of Oral & Maxillofacial Surgery, West China Hospital of Stomatology, Sichuan University, Chengdu 610041, P.R. China; 2 State Key Laboratory of Oral Diseases, Sichuan University, Chengdu 610044, P.R. China; 3 Guanghua School of Stomatology, Sun Yat-sen University, Guangzhou 510055, P.R.China

**Keywords:** Chronic suppurative osteomyelitis of the jaw, Recurrence, Life-threatening complications, Risk factors, Logistic regression analysis

## Abstract

**Background:**

Clinically, recurrence and life-threatening complications are challenging problems for chronic suppurative osteomyelitis of the jaw (CSOJ), but there is no quantitative analysis or report about the causes of or risk factors for the two problems to date. Doctors identify the causes or risk factors only through clinical experience. We performed a retrospective study of 322 patients with CSOJ to quantificationally analysed the risk factors for the abovementioned two problems by logistic regression analysis.

**Methods:**

A retrospective study of 322 patients hospitalized with CSOJ was performed. The socio-demographic and clinical characteristics were recorded. The risk factors for the above two problems were analyzed by logistic regression analysis. Frequency and percentage were used to indicate descriptive research factors. A univariate logistic regression analysis was performed to calculate the odds ratio (OR) and to identify independent risk factors. The independent risk factors were further identified by multivariate logistic regression analysis.

**Results:**

An age from 6–12 years or > 65 years, pre-admission antibiotic administration, a lesion at the mandibular ramus, concurrent maxillofacial space infection (MSI), and conservation of pathogenic teeth were found to be risk factors for recurrence. An age > 65 years, admission temperature > 39 degree Celsius, admission white blood cell (WBC) count >15×10^9^/L, pre-admission antibiotic administration, concurrent MSI, pre-existing diabetes, and respiratory difficulty were found to be risk factors for life-threatening complications.

**Conclusions:**

The results indicate that doctors should remain mindful of the risk factors listed above, and the management of CSOJ should be increasingly aggressive when the above risk factors are present, especially when the lesion is located at the mandibular ramus. In addition, pathogenic teeth must be extracted, and antibiotics should be administered properly.

**Trial registration:**

Clinicaltrials.gov (NCT01670422)

## Background

Osteomyelitis is an inflammatory condition of bone and bone marrow, which has a tendency to involve the adjacent cortex, periosteum, and soft tissue [1]. Chronic supperative Osteomyelitis of the jaw (CSOJ) is a persistent inflammatory process in the mandible or the maxilla presenting with necrosis of mineralized and marrow tissues, suppuration, resorption, sclerosis, and hyperplasia [2]. This is mainly triggered by inoculation of micro-organisms into the jawbones as a result of trauma or odontogenic infection [3]. Some other reasons such as steroids, chemotherapeutic agents, and biphosphonates such as alendronic acid are also linked to CSOJ [3].

In recent decades, CSOJ has decreased significantly following the widespread use of broad-spectrum antibiotics and improved dental care, especially in developed countries. However, there are still many people who suffer from CSOJ in developing countries. These people with CSOJ are mostly recognized as a low income population, who not only receive poor healthcare because of affordability problems, but also receive lower health system coverage [4]. Meanwhile, the oral health knowledge and oral hygiene of people in developing countries are poor because of the limited oral health education, thus they easily suffer from dental diseases. But worse still, as soon as they feel pain or swelling in oral and maxillofacial region, they usually take antibiotics and/or anodyne without consultation, rather than go to doctor for help [5,6]. Therefore, incidence of CSOJ in developing countries is still relatively high in nowadays.

The acknowledged and effectual treatment for CSOJ is a combination of antimicrobial therapy and surgery consisting of incision and drainage, debridement or sequestrectomy [7]. Recently, some scholars have advocated the use of adjunctive treatment such as hyperbaric oxygen, which purportedly has a good short-term clinical effect [8,9]. However, some problems, which include high recurrence rate and potential life-threatening complications, remain challenges for oral surgeons.

Recurrence of CSOJ not only seriously influences the lives and jobs of the patients but also results in physiological and psychosocial disturbances because of facial malformation [10]. Thus, many scholars have investigated the issue of recurrence. Reichart et al. reported that recurrence was related to the complexity of the operation, which is due to the complicated anatomic structure of the oral and maxillofacial region [11]. Becconsall-Ryan et al. and Ozdemir et al. reported that infectious diseases associated with CSOJ, such as caries, periodontitis and periapical inflammation, were related to recurrence [12,13]. However, there remains no conclusive research defining the risk factors for CSOJ recurrence. Thus, doctors identify the risk factors only through clinical experience.

Although the treatment of CSOJ is effective and the incidence has dropped significantly in the past few decades. However, CSOJ remains a potentially lethal infection because of the possibility of life-threatening complications, such as sepsis [14], brain abscess [15], suppurative jugular thrombophlebitis [16], carotid erosion [16], and respiratory obstruction [16]. Ronai et al. reported a case in which the extraction of a tooth led to the acute exacerbation of existing chronic suppurative osteomyelitis of the jaw and then to phlegmon and ultimately sepsis. Finally, the patient died due to multi-organ failure [14]. Karshiev et al. found the mortality of CSOJ to be 0.56% [15]. The major causes are thrombosis of the cavernous sinus, abscess of the brain and sepsis. Reynolds et al. found that maxillofacial infections could spread to the fascial spaces of the lower head and upper neck, resulting in cavernous sinus thrombosis, suppurative jugular thrombophlebitis or carotid erosion [16]. However, it is a pity that there is still no research specialized in risk factors analysis of life-threatening complications for CSOJ.

Therefore, the objective of this study is devoted to appraising the potential risk factors of recurrence and life-threatening complications of CSOJ and providing referential basis for clinical practice.

## Methods

### Participants

Patients with a diagnosis of CSOJ who were admitted to our hospital from 1980 to 2009 were investigated. The diagnostic criteria on admission were made on the basis of the following three points: (1) The presence of sequestra and laminations of periosteal new bone in the pathological area, which were presented by conventional radiography or computerized tomography. (2) The positive pathological micro-organisms culture and (or) chronic inflammatory changes in bone biopsy. (3) Symptoms such as local pain, pyorrhea, fever, swelling, fistula, neuropalsy, odontoseisis, lymphadenopathy, bromopnea, and trismus served as assistant diagnostic criteria. Criteria of discharge from hospital include three aspects as below: (1) Sequestra are curetted or resected. (2) Symptoms of CSOJ disappear or ease obviously. (3) The results of pathological micro-organisms culture are negative. Patients without confirmed evidence of CSOJ were excluded. Paget’s disease, hypercementosis, fibrous dysplasia, and early stage malignant bone tumor were differentially diagnosed and excluded. Ultimately, 322 patients were enrolled in the study of life-threatening complications. Patients without standard treatment (a combination of antimicrobial therapy and surgery consisting of incision and drainage, debridement or sequestrectomy) and patients without follow-up records or with less than 1 year of follow-up were excluded. A total of 276 patients were enrolled in the study of disease recurrence. Patients were divided into four classes according to their age (years): <6, 6–12, 13–65, >65. Due to the retrospective nature of this study, it was approved by the institutional review board of Sichuan University for scientific and ethical integrity.

### Data collection and analysis

The characteristics studied were age, sex, occupation, admission temperature, admission WBC count, pre-admission antibiotic administration, pre-existing diabetes, the location of the lesion, treatment, the length of stay, the recurrence rate, complications and prognosis. Frequency and percentage were used to indicate descriptive research factors. A univariate logistic regression analysis was performed to calculate the odds ratio (OR) and to identify independent risk factors. A value of p< 0.05 or p< 0.01 was considered statistically significant. The significant risk factors were further identified by multivariate logistic regression analysis. All the data were analyzed using SPSS version 17.0 (SPSS Inc., Chicago, USA).

## Results

**Figure 1:**
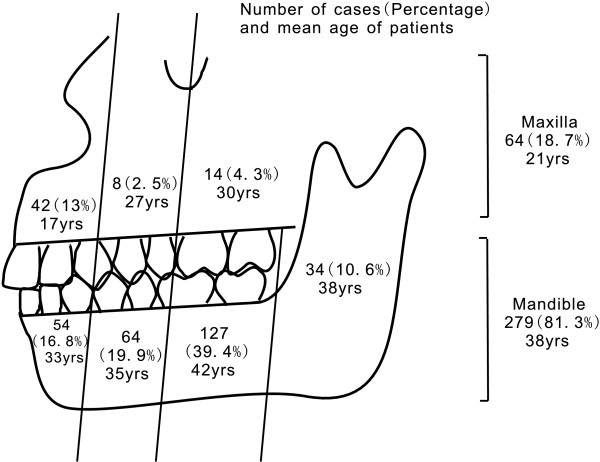
The lesion distribution and the mean patient age.

A total of 476 patient records were arranged, and 322 qualified cases were enrolled in this study, including 185 males (57.5%) and 137 females (42.5%), with an age range of 1 month to 83 years (median 32 years). 154 cases were excluded because of lack of confirmed evidence listed in Method. There were 258 cases (80.1%) with disease confined to the mandible, 43 cases (13.4%) with disease confined to the maxilla, and 21 cases (6.5%) with disease in both the maxilla and mandible. The lesion distribution and mean age of patients are presented in Figure 1. Among the 322 cases, 276 cases had complete chart records and meet the requirements of disease recurrence study. Patients without standard treatment (a combination of antimicrobial therapy and surgery consisting of incision and drainage, debridement or sequestrectomy) and patients without follow-up records or with less than 1 year of follow-up were excluded.

**Figure 2:**
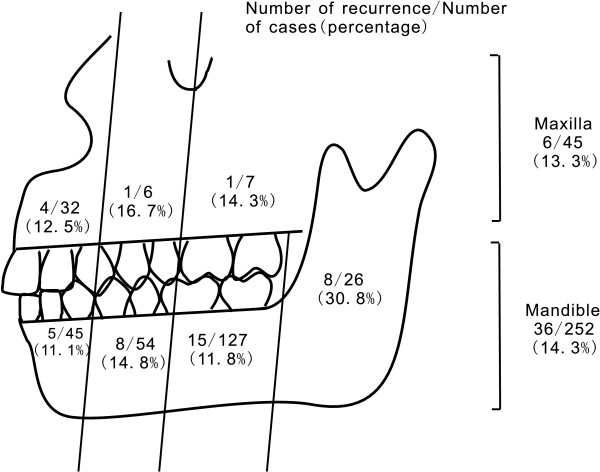
The recurrence rate distribution.

A total of 42 patients recurred (15.2%) within a year after treatment. The recurrence rate distribution is shown in Figure 2. In a univariate logistic regression analysis, pre-admission antibiotic administration, a lesion at the mandibular ramus, concurrent MSI and conservation of pathogenic teeth were found to be risk factors for disease recurrence. These factors remained important in a multivariate model. Furthermore, an age from 6–12 years or > 65 years was also found to be a risk factor for disease recurrence in the multivariate model. The details are shown in Table 1.

**Table 1:** Risk factors for recurrence of chronic suppurative osteomyelitis of the jaw

**Variable**	**No. of recurrence/Total no. of patients (%)**	**Univariate analyses**		**Multivariate analyses**	
		**OR(95% CI)**	**P**	**OR(95% CI)**	**P**
Age					
<6	3/24 (12.5)	0.31(0.07-1.46)	0.138	0.05(0.01-0.31)	0.002*
6-12	1/4 (25.0)	0.72(0.06-8.46)	0.796	0.11(0.01-1.63)	0.109
13-65	32/229 (14.0)	0.35(0.13-0.99)	0.048*	0.09(0.02-0.37)	0.001*
>65	6/19 (31.6)	1(ref.)^#^		1(ref.)	
Sex					
Male	26/165 (15.8)	1.11(0.57-2.18)	0.761	1.57(0.71-3.49)	0.270
Female	16/111 (14.4)	1(ref.)		1(ref.)	
Occupation					
Farmer	29/168 (17.3)	1.36(0.29-6.34)	0.699	0.77(0.15-3.80)	0.743
Worker	5/42 (11.9)	0.88(0.15-5.09)	0.885	0.35(0.05-2.76)	0.319
Student	3/25 (12.0)	0.89(0.13-6.02)	0.902	0.89(0.13-6.02)	0.902
Staff	2/15 (13.3)	1.00(0.12-8.21)	1.000	1.00(0.12-8.21)	1.000
Cadre	1/11 (9.1)	0.65(0.05-8.23)	0.739	0.67(0.06-7.05)	0.736
Other	2/15 (13.3)	1(ref.)		1(ref.)	
PAAA					
Yes	32/158 (20.3)	2.74(1.29-5.84)	0.009*	6.52(2.21-19.28)	0.001*
No	10/118 (8.5)	1(ref.)		1(ref.)	
Location	8/26 (30.8)	3.56(1.02-12.39)	0.046*	8.58(2.08-35.31)	0.003*
Mandibular ramus	2/13 (15.4)	1.46(0.25-8.54)	0.678	3.25(0.59-18.06)	0.178
Posterior maxilla	4/32 (12.5)	0.57(0.14-2.26)	0.425	3.43(0.78-15.15)	0.104
Anterior maxilla	23/181 (12.7)	1.69(0.60-4.74)	0.320	2.38(0.88-6.40)	0.087
Posterior mandible	5/45 (11.1)	1(ref.)		1(ref.)	
Anterior mandible					
Concurrent MSI	17/54 (32.4)	3.62(1.78-7.36)	0.000*	2.73(1.23-6.03)	0.013*
Yes	25/222 (11.3)	1(ref.)		1(ref.)	
No					
Conservation of PT	11/34 (32.4)	3.49(1.11-12.21)	0.000*	7.21(3.23-21.48)	0.001*
Yes	26/204 (12.7)	1(ref.)		1(ref.)	
No					

#: Set the last item of each class as a reference with the odds ratio for “1” in Logistic regression analysis.

*: P < 0.05.

Key to the abbreviations:

No.: Number; OR: Odds ratio; P: P-value; CI: confidence interval; PAAA: pre-admission antibiotic administration; MSI: Maxillofacial space infection; PT: pathogenic teeth; ref.: reference.

In total, 31 patients (9.6%) had life-threatening complications, and 3 died (0.9%). In a univariate analysis, high admission WBC count, high admission temperature, pre-admission antibiotic administration, concurrent MSI, pre-existing diabetes, and respiratory difficulty were found to be risk factors for life-threatening complications. These factors remained statistically significant in a multivariate model. Furthermore, an age > 65 years, admission temperature > 39 degree Celsius, and an admission WBC count >15×10^9^/L were found to be the risk factors for life-threatening complications in the multivariate model. The detailed results are shown in Table 2.

**Table 2:** Risk factors for life-threatening complications of chronic suppurative osteomyelitis of the jaw

**Variable**	**No. of recurrence/Total no. of patients (%)**	**Univariate analyses**		**Multivariate analyses**	
		**OR(95% CI)**	**P**	**OR(95% CI)**	**P**
Age					
<6	5/34 (14.8)	0.78(0.18-3.28)	0.730	0.12(0.02-0.82)	0.030*
6-12	0/7				
13-65	22/259 (8.5)	0.42(0.13-1.34)	0.143	0.11(0.02-0.55)	0.007*
>65	4/22 (18.2)	1(ref.)^#^		1(ref.)	
Sex					
Male	17/185 (9.2)	0.89(0.42-1.87)	0.757	0.96(0.40-2.30)	0.955
Female	14/137 (10.2)	1(ref.)		1(ref.)	
Occupation					
Farmer	23/178 (12.9)	0.93(0.30-2.91)	0.897	0.45(0.12-1.66)	0.232
Worker	2/51 (3.9)	0.26(0.04-1.49)	0.129	0.27(0.05-1.59)	0.272
Student	1/31 (3.2)	0.21(0.02-1.99)	0.173	0.34(0.11-2.21)	0.231
Staff	1/19 (5.3)	0.35(0.04-3.37)	0.362	0.42(0.09-3.42)	0.421
Cadre	0/14				
Other	4/29 (13.8)	1(ref.)		1(ref.)	
Temperature					
36-37.5°C	5/123 (4.1)	0.06(0.02-0.19)	0.000*	0.12(0.04-0.39)	0.001*
37.6-38°C	3/74 (4.1)	0.06(0.01-0.23)	0.000*	0.13(0.03-0.50)	0.003*
38.1-39°C	4/59 (6.8)	0.10(0.03-0.35)	0.000*	0.21(0.06-0.76)	0.017*
39.1-40°C	9/43 (20.9)	0.34(0.11-1.04)	0.058	0.61(0.19-1.92)	0.393
>40°C	10/23 (43.5)	1(ref.)		1(ref.)	
WBC					
<10×10^9^/L	6/113 (5.3)	0.17(0.06-0.44)	0.000*	0.04(0.01-0.28)	0.002*
10×10^9^/L −15×10^9^/L	5/130 (3.8)	0.12(0.04-0.33)	0.000*	0.10(0.04-0.30)	0.000*
>15×10^9^/L	20/79 (25.3)	1(ref.)		1(ref.)	
PAAA					
Yes	24/178 (13.5)	3.05(1.27-7.30)	0.012*	3.35(1.36-8.26)	0.009*
No	7/144 (4.9)	1(ref.)		1(ref.)	
Concurrent MSI					
Yes	16/74 (21.6)	4.29(2.00-9.17)	0.000*	6.90(2.20-21.62)	0.001*
No	15/248 (6.0)	1(ref.)		1(ref.)	
PED					
Yes	17/58 (29.3)	7.40(3.39-16.16)	0.000*	16.17(4.51-58.04)	0.000*
No	14/264 (5.3)	1(ref.)		1(ref.)	
Concurrent RD					
Yes	18/31 (58.1)	29.61(11.98-73.16)	0.000*	39.32(12.55-123.21)	0.000*
No	13/291 (4.5)	1(ref.)		1(ref.)	

#: Set the last item of each class as a reference with the odds ratio for “1” in Logistic regression analysis.

*: P < 0.05.

Key to the abbreviations:

No.: Number; OR: Odds ratio; P: P-value; CI: confidence interval; WBC: White blood cells; PAAA: pre-admission antibiotic administration; MSI: Maxillofacial space infection; PED: pre-existing diabetes; RD: respiratory difficulty; ref.: reference.

## Discussion

People with CSOJ get great suffering. Clinically, recurrence and life-threatening complications are two major thorny problems for some patients. Most of these patients encounter damaged stomatognathic system or facial deformity, which result in serious physiological and psychological disorders [10]. Hence it is a pertinent time to appraise the reason of recurrence and life-threatening complications of CSOJ.

This study showed that age, admission WBC count, admission temperature, lesion location, pre-admission antibiotic administration, concurrent MSI, pre-existing diabetes and respiratory difficulty before admission were closely related to the two challenging clinical issues of CSOJ.

An age from 6–12 years or > 65 years was a risk factor for recurrence, and an age > 65 years was a risk factor for life-threatening complications. Ozkan et al. found that physical status, such as general health, dental status, oral hygiene practices and denture hygiene, is closely related to the worsening or relapse of infectious disease in the oral and maxillofacial region [17]. An age > 65 years represents the elderly, who have a low immune functions and a special oral environment. Residual roots and crowns are common for the elderly. Therefore, as this study shows, the elderly potentially increase the risk of developing life-threatening complications and recurrence. As for an age from 6–12 years, the patients are in a mixed dentition period. Some papers report that there are more pathogenic bacteria in mixed dentition than in deciduous dentition and permanent dentition. Umeda et al. investigated the oral microorganisms in the oral cavities of children from 1–15 years of age and found that there were more pathogenic bacteria in mixed dentition than in deciduous or permanent dentition [18]. The high recurrence rate of CSOJ in patients between the ages of 6 and 12 years may be related to the local oral environment in the mixed dentition period, but it needs further research. Stated thus, it may be concluded that the age of patients is related to the recurrence and life-threatening complications. Doctors should remain mindful of these risks when treating patients with CSOJ who are between 6 years and 12 years, or >65 years old.

The results demonstrated that a high admission WBC count and temperature are related to life-threatening complications. Both WBC count and temperature correlate positively with the severity of infection. A WBC count exceeding 15×10^9^/L or a temperature exceeding 39 degree Celsius usually indicates a severe infection, which may itself be a life-threatening complication [19]. Doctors should remain mindful of the temperature and WBC count to prevent life-threatening complications.

Maxillofacial space infection (MSI) refers to infections in the potential spaces and fascial planes of the maxillofacial region. It usually presents as an acute inflammatory process. The main causes are odontogenic infection, lymphadenitis, and trauma [20]. This study found that CSOJ accompanied by MSI was related to the two issues. CSOJ and MSI are interrelated. They are capable of acting on or influencing each other. MSI is an infectious disease of loose soft tissue. So it is prone to diffuse, and some of the infectious tissue may remain after treatment. When immunity is reduced, CSOJ occurs again, leading to an increased rate of disease recurrence. The risk of recurrence for patients with concurrent MSI was about four times that for patients without MSI (OR: 3.62:1). Among the patients with concurrent MSI, 16 cases had life-threatening complications (21.6%). The risk of life-threatening complications for patients with concurrent MSI was 3.6 times that for patients without MSI (6.0%). Among the 16 cases with life-threatening complications, 8 cases had concurrent submandibular space infection, 5 had concurrent parapharyngeal space infection, and 3 had concurrent cellulitis of the floor of the mouth. These maxillofacial space infections often cause airway obstruction, even asphyxia [18]. Some of the patients with a severe upper respiratory MSI may present with respiratory difficulty on admission. The statistical data showed that 58.1% (18/31) of the patients with respiratory difficulty on admission [subjective labored breathing and objective increased respiratory rate (> 20 per min)] presented with respiratory obstruction (asphyxia caused by tissue swelling). Therefore, Attention should be paid to the patients with concurrent MSI (especially submandibular space infection, parapharyngeal space infection and cellulitis of the floor of the mouth) and patients who present with respiratory difficulty.

The results showed that pre-existing diabetes was related to life-threatening complications. Among the 58 patients with diabetes on admission, 17 (29.3%) had life-threatening complications, compared to 5.3% of those without diabetes. High blood glucose of patients with diabetes leads to increased glucose content in the various bodily fluids, which facilitates bacterial growth and reproduction. Those factors make patients vulnerable to infection [21]. Therefore, for patients with diabetes on admission, it is essential to monitor blood glucose and maintain it within the range of normal values. Other potential confounders such as hyperlipemia and autoimmune disease are exactly relevant to prognosis of CSOJ. But be confined to the poor integrality of these data and haziness of some diseases’ definitions, especially to the statistical method, those comorbidities were not included in this study.

This study showed that the location of the lesion is closely related to disease recurrence. The recurrence rate for patients whose lesion was located at the mandibular ramus was 30.8% (8/26), concentrating on the neck of the condylar process (4/11, 36.4%), the mandibular notch (2/9, 22.2%), and the lingual side of mandibular ramus (2/6, 33.3%), whereas it was 15.4% (2/13) at the posterior maxilla, 12.5% (4/32) at the anterior maxilla, 12.7% (23/181) at the posterior mandible, and 11.1% (5/45) at the anterior mandible. This difference may be related to the complicated anatomic structure of and difficult operative approach to the mandibular ramus region, in accordance with previous reports [11]. We found that the recurrence rate of patients in whom pathogenic teeth were conserved (11/34, 32.4%) was significantly higher than that of others (26/204, 12.7%). This demonstrated the importance of simultaneous extraction of pathogenic teeth [22]. More aggressive surgical treatment should be recommended for CSOJ, particularly with lesions located at the neck of the condylar process, the mandibular notch, and the lingual side of the mandibular ramus. In addition, pathogenic teeth must be extracted simultaneously.

At present, the abuse of antibiotics is a universal problem [23]. Long-term abuse of antibiotics results in drug resistance, gradually increasing the challenges of treating infectious diseases [24,25]. This study showed that the recurrence rate and incidence of life-threatening complications for patients with pre-admission antibiotic administration were significantly higher. In total, 178 patients (55.3%) had pre-admission antibiotics without consultation, and 158 of these patients were included in our study of disease recurrence. Among them, 24 patients (13.5%) had life-threatening complications, and 32 patients (20.3%) had disease recurrence. For patients without pre-admission antibiotic administration, only 4.9% had life-threatening complications, and 8.5% had disease recurrence. Pre-admission antibiotic administration without consultation leads to bacterial resistance to antibiotics, allowing residual bacteria to remain, which increases the risk of CSOJ recurrence when the patient’s immunity reduces [25]. The time from the onset of symptoms to presentation for those who had pre-admission antibiotic administration was longer than that for those who did not receive antibiotics. This delay in presentation may itself increase the risk of life-threatening complications. Therefore, proper antibiotic treatment is essential to minimize the recurrence rate and the incidence of life-threatening complications.

## Conclusions

In summary, the results of this study indicate that more attention should be paid to the risk factors, including an age between 6 years and 12 years, or > 65 years old, a high admission WBC count, a high admission temperature, a lesion located at the mandibular ramus, pre-admission antibiotic administration, concurrent MSI, pre-existing diabetes and respiratory difficulty at the time of presentation. More aggressive surgical treatment should be recommended for CSOJ, particularly for lesions located at the neck of the condylar process, the mandibular notch, and the lingual side of the mandibular ramus. In addition, pathogenic teeth must be extracted, and antibiotics should be administered properly.

## Competing interests

The authors declare that they have no competing interests.

## Authors’ contributions

LL and CL designed the study. LC, TL, CL and LL, wrote the initial draft and vouch for the data, analysis, interpretation, and manuscript submission. LC and TL provided guidance on data modeling and contributed to writing. WJ, WT and WT, supervised the study and contributed to analysis. All authors contributed to data collection and writing of the report and all approved the final draft.

## Pre-publication history

The pre-publication history for this paper can be accessed here:

http://www.biomedcentral.com/1471-2334/13/313/prepub

## References

[B1] Noel W Lin GY Michael K Modern Surgical Pathology 2009 2 Philadelphia: Saunders

[B2] Cawson RA Essentials of dental surgery and pathology 1984 4 Edinburgh: Churchill Livingstone

[B3] Peterson LJ Ellis E Hupp JR Contemporary oral and maxillofacial surgery 2003 CV, Mosby: St. Louis

[B4] Liu Y Rao K Wu J Gakidou E China’s health system performance Lancet 2008 372 1914 1923 10.1016/S0140-6736(08)61362-8 18930536

[B5] Wang H Zhang L Hsiao W Ill health and its potential influence on household consumptions in rural China Health Policy 2006 78 167 177 10.1016/j.healthpol.2005.09.008 16263191

[B6] Lin HC Schwarz E Oral health and dental care in modern-day China Community Dent Oral Epidemiol 2001 29 319 328 10.1034/j.1600-0528.2001.290501.x 11553104

[B7] van Merkesteyn JP Groot RH van den Akker HP Bakker DJ Borgmeijer-Hoelen AM Treatment of chronic suppurative osteomyelitis of the mandible Int J Oral Maxillofac Surg 1997 26 450 454 10.1016/S0901-5027(97)80012-4 9418149

[B8] Urade M Noguchi K Moridera K Hashitani S Kishimoto H Diffuse sclerosing osteomyelitis of the mandible treated with hyperbaric oxygen and pamidronate: a long-term follow-up Int J Oral Maxillofac Surg 2009 38 566 10.1016/j.oooo.2012.02.017 22771405

[B9] Adams JR Bryant DG Cranial osteomyelitis: a late complication of a dental infection Br J Oral Maxillofac Surg 2008 46 673 674 10.1016/j.bjoms.2008.03.025 18486999

[B10] Hudson JW Osteomyelitis of the jaws: a 50-year perspective J Oral Maxillofac Surg 1993 51 1294 1301 10.1016/S0278-2391(10)80131-4 8229407

[B11] Reichart PA Philipsen HP Rateitschak KH, Wolf HF Osteomyelitis Oralpathologie 1999 Stuttgart: Thieme 191 195

[B12] Becconsall-Ryan K Tong D Love RM Radiolucent inflammatory jaw lesions: a twenty-year analysis Int Endod J 2010 43 859 865 10.1111/j.1365-2591.2010.01751.x 20738428

[B13] Ozdemir A Guven G Dilsiz A Sencimen M Diagnosis and treatment of mandibular extraoral sinus of periodontal origin in a 9-year-old boy: a case report J Indian Soc Pedod Prev Dent 2008 26 Suppl 3 76 78 19075453

[B14] Ronai A Olasz L Muhl D Lethal complication of an odontogenic infection developing after tooth extraction in a patient with untreated diabetes Case report. Fogorv Sz 2001 94 27 31 11262799

[B15] Karshiev KK An analysis of the mortality of patients with suppurative-inflammatory diseases of the maxillofacial area Stomatologiia 1997 76 9 10 9411944

[B16] Reynolds SC Chow AW Life-threatening infections of the peripharyngeal and deep fascial spaces of the head and neck Infect Dis Clin North Am 2007 21 557 576 10.1016/j.idc.2007.03.002 17561083

[B17] Ozkan Y Ozcan M Kulak Y Kazazoglu E Arikan A General health, dental status and perceived dental treatment needs of an elderly population in Istanbul Gerodontology 2011 28 28 36 10.1111/j.1741-2358.2010.00363.x 21320160

[B18] Umeda M Miwa Z Takeuchi Y Ishizuka M Huang Y Noguchi K Tanaka M Takagi Y Ishikawa I The distribution of periodontopathic bacteria among Japanese children and their parents J Periodontal Res 2004 39 398 404 10.1111/j.1600-0765.2004.00754.x 15491344

[B19] Horseman MA Surani S A comprehensive review of Vibrio vulnificus: an important cause of severe sepsis and skin and soft-tissue infection Int J Infect Dis 2011 15 157 166 10.1016/j.ijid.2010.11.003 21177133

[B20] Jerry EB Susan M Hiromasa N Naggar EL, Adel K Lesions of the Oral Cavity Diagnostic Surgical Pathology of the Head and Neck 2009 Philadelphia: Lippincott Williams & Wilkins 191 308

[B21] Gupta S Koirala J Khardori R Khardori N Infections in Diabetes Mellitus and Hyperglycemia Infect Dis Clin North Am 2007 21 617 638 10.1016/j.idc.2007.07.003 17826615

[B22] Rice S Bridle C The management of patients with oral and maxillofacial infections: applying the evidence to clinical practice Br J Oral Maxillofac Surg 2009 47 50 10.1016/j.bjoms.2008.03.021 18490088

[B23] English BK Gaur AH The use and abuse of antibiotics and the development of antibiotic resistance Adv Exp Med Biol 2010 659 73 82 10.1007/978-1-4419-0981-7_6 20204756

[B24] Graffunder EM Venezia RA Risk factors associated with nosocomial methicillin-resistant Staphylococcus aureus (MRSA) infection including previous use of antimicrobials J Antimicrob Chemother 2002 49 999 1005 10.1093/jac/dkf009 12039892

[B25] Pigrau C Almirante B Rodriguez D Larrosa N Bescos S Raspall G Pahissa A Osteomyelitis of the jaw: resistance to clindamycin in patients with prior antibiotics exposure Eur J Clin Microbiol Infect Dis 2009 28 317 323 10.1007/s10096-008-0626-z 18797941

